# Enhanced Intestinal Permeability of Cefixime by Self-Emulsifying Drug Delivery System: In-Vitro and Ex-Vivo Characterization

**DOI:** 10.3390/molecules28062827

**Published:** 2023-03-21

**Authors:** Arshad Mahmood, Laraib Khan, Muhammad Ijaz, Imran Nazir, Mahrukh Naseem, Muhammad Azam Tahir, Muhammad Naeem Aamir, Masood Ur Rehman, Mulazim Hussain Asim

**Affiliations:** 1 College of Pharmacy, Al Ain University, Abu Dhabi Campus, Abu Dhabi P.O. Box 112612, United Arab Emirates; 2 Health and Biomedical Research Centre (HBRC), Al Ain University, Abu Dhabi P.O. Box 112612, United Arab Emirates; 3 Riphah Institute of Pharmaceutical Sciences, Riphah International University, Islamabad 44000, Pakistan; 4 Department of Pharmacy, COMSATS University Islamabad, Lahore Campus, Lahore 54000, Pakistan; 5 Department of Zoology, University of Baluchistan, Quetta 87300, Pakistan; 6 Department of Pharmacy, Khalid Mahmood Institute of Medical Sciences, Sialkot 51310, Pakistan; 7 Department of Pharmaceutics, Faculty of Pharmacy, The Islamia University of Bahawalpur, Bahawalpur 63100, Pakistan; 8 College of Pharmacy, University of Sargodha, Sargodha 40100, Pakistan

**Keywords:** oral bioavailability, cefixime, self-nanoemulsifying drug delivery system (SEDDS), permeation enhancement

## Abstract

Background: Cefixime (CFX) belongs to a group of third-generation cephalosporin antibiotics with low water solubility and low intestinal permeability, which ultimately leads to significantly low bioavailability. Aim: This study aimed to increase solubility, improve drug release, and intestinal permeability of CFX by loading into SEDDS. Methods: Suitable excipients were selected based on drug solubility, percent transmittance, and emulsification efficiency. Pseudo-ternary phase diagram was fabricated for the identification of effective self-emulsification region. The best probably optimized formulations were further assessed for encumbered drug contents, emulsification time, cloud point measurement, robustness to dilution, mean droplet size, zeta potential, polydispersity index (PDI), and thermodynamic and chemical stability. Moreover, in vitro drug release studies and ex vivo permeation studies were carried out and apparent drug permeability P*_app_* of different formulations was compared with the marketed brands of CFX. Results: Amongst the four tested SEDDS formulations, F-2 formulation exhibited the highest drug loading of 96.32%, emulsification time of 40.37 ± 3 s, mean droplet size of 19.01 ± 1.12 nm, and demonstrated improved long-term thermodynamic and chemical stability when stored at 4 °C. Release studies revealed a drug release of 97.32 ± 4.82% within 60 min in simulated gastric fluid. Similarly, 97.12 ± 5.02% release of CFX was observed in simulated intestinal fluid within 120 min; however, 85.13 ± 3.23% release of CFX was observed from the marketed product. Ex vivo permeation studies displayed a 2.7-fold increase apparent permeability compared to the marketed product in 5 h. Conclusion: Owing to the significantly improved drug solubility, in vitro release and better antibacterial activity, it can be assumed that CFX-loaded SEDDS might lead to an increased bioavailability and antibacterial activity, possibly leading to improved therapeutic effectiveness.

## 1. Introduction

Medication given orally are considered the easy and expedient route of drug delivery, as it is the safer, convenient, economical, and non-invasive method of drug administration [1]. However, oral administration of the drugs is associated with various challenging barriers that are being responsible for low bioavailability. These barriers must be overcome using various carrier systems in order to augment the bioavailability of drugs [2,3]. Oral absorption and bioavailability of drugs are also dependent on various factors such as solubility, dissolution and permeation across the intestinal membranes [4].

Cefixime (CFX), a third generation antibiotic, is widely utilized in the mitigation of innumerable infections caused by Gram-negative and Gram-positive bacteria such as respiratory and urinary tract infections, acute otitis media and gonococcal urethritis [5]. The minimum inhibitory concentration for CFX against Escherichia coli is 0.75 µg/mL and for standard *Staphylococcus aureus*, it is 8 µg/mL [6]. However, as per the biopharmaceutical classification system (BCS), CFX belongs to class IV drugs, exhibiting poor water solubility and less permeability across the absorption membrane when given orally [7]. The oral bioavailability of CFX reported in the literature ranged from 30 to 50% only, with the lower range for solid dosage forms such as tablets and the higher range for liquids such as solutions [8]. Therefore, high doses are often required, especially in cases of *Staphylococcus aureus* infections, to achieve the therapeutics outcomes and minimize the chances of resistance. Numerous approaches such as gastro-retentive, pH sensitive hydrogels, muco-adhesive nanoparticles and mucilage were employed in order to improve the oral bioavailability of CFX [9,10,11,12]. However, all those strategies appear very complicated in terms of industrial manufacturing. A multi-step synthesis/conjugation of the starting material with compromised yield may be considered an option when basic and fundamental systems have failed. It is widely known that an industrial manufacturing process with a fewer number of steps, less equipment and a high yield is considered ideal.

Among the lipid-based dosage forms, self-emulsifying drug delivery systems (SEDDS) possess the advantage of simple manufacturing and an easy scale-up at an industrial level compared to all nano-particulate drug delivery systems [13]. SEDDS are simply homogeneous blends of surfactants, oils and co-surfactants; and the anhydrous nature of prepared oily mixtures allows them to be incorporated into soft gelatin capsules. The mixture will form oil in water (O/W) emulsion upon dilution with aqueous media of stomach or intestine [14]. Importantly, these lipid-based dosage forms have capacity to not only resolve the solubility issue but after dilution to micro- and nano-emulsion, have the tendency to overcome different oral barriers and improve the permeation of the lipophilic drugs across the mucosa [15,16]. Katherina et al, in a recent article, demonstrated improved solubility of cannabidiol linked with enhanced oral bioavailability when incorporated within SEDDS. It was observed that the increased solubility of cannabidiol in the aqueous environment of the intestinal tract could be main reason for enhancing the oral bioavailability [17].

Therefore, the current study aimed to develop and optimize CFX-loaded SEDDS in order to improve the solubility and permeability across the intestinal membrane that can provide a higher bioavailability. An increase in the oral absorption of CFX is desired in many aspects from a pharmacokinetic point of view; for instance, the onset of action will be rapid through an increase in the rate of absorption, a higher Cmax with same dose through increase in the bioavailability and a prolonged duration of action for the drug. Collectively, all these parameters may lower the chances of drug resistance. In order to achieve the aim, drug solubility will be assessed in various oils, surfactant and then, optimized SEDDS formulation will be prepared. CFX-loaded SEDDS would be evaluated for droplet size, zeta potential and dissolution profile. CFX loaded oily droplets will be further assessed for ex vivo permeability and antibacterial potential.

## 2. Results and Discussion 

### 2.1. Screening of Various Excipients Based on Solubility Studies

**Figure 1:**
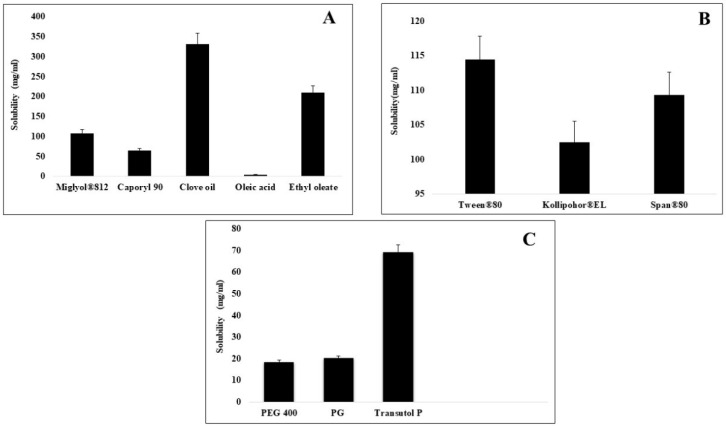
Comparative solubility of cefixime in various oils (**A**), surfactants (**B**), and co-surfactants (**C**) evaluated at 25 °C.

A formulation having a self-emulsifying ability can lead to drug precipitation upon dilution into lumen of gut. So, the selection of appropriate excipients is necessary for the selected drug, which, upon aqueous dilution, can retain the drug in solubilized form [18]. The purpose of solubility studies was to choose an appropriate oil, surfactant and co-surfactant for the preparation of CFX-incorporated SEDDS having maximum solubility capacity, which will also increase the drug loading [19]. The solubility of CFX was studied in different excipients such as oils, surfactants and co surfactants, the results of which are given in Figure 1. Amongst oils, the highest solubility of CFX was in clove oil, which was 331.51 ± 0.15 mg/mL. Among surfactants, Kolliphore EL displayed the maximum drug dissolving capability of 69.43 ± 0.22 mg/mL. Clove oil was chosen as oil phase for further studies based on its property of highest solubility for a drug [20]. Clove oil is an essential oil that is on the list of, what are generally regarded as, safe chemicals. The antibacterial activity of clove oil was also reported in the literature, and it can augment the antibacterial activity of CFX [21].

### 2.2. Screening of Various Excipients Based on Emulsification Ability

#### 2.2.1. Screening of Surfactants 

At ambient temperature, a self-emulsification system exhibited a clear and homogeneous liquid [19]. The selection of the suitable SEDDS excipients should be on the basis of their emulsification properties rather than their drug solubilization capabilities [22]. Non-ionic surfactants used are safer, stable and biocompatible, and less affected by pH and ionic strength in a medium. They are more preferable for oral ingestion because of low critical micelle concentrations [23]. The highest number of inversions and lowest % transmittance was shown by Span 80 in ethyl oleate 62.15 ± 29.32% and clove oil 69.32 ± 0.11%, respectively, with turbid appearance. The lowest number of inversions and highest % transmittance was shown by the ethyl oleate-Tween 80 combination 98.33 ± 0.27% and clove oil-Tween 80 combination 96.29 ± 0.16% having a clear appearance (Table 1). Tween 80 was chosen because it was better mixable with the drug. It exhibited the highest emulsification with clove oil, and it has high HLB value. The surfactants having greater HLB values can form more stable nano-emulsions [24].

#### 2.2.2. Screening of Cosurfactants

Co-surfactants are also required for the support of surfactants in achieving low fluidization of the hydrocarbon region and interfacial tension present in interfacial films, which is seldom attained by surfactant alone [23]. In a formulation, the use of co-surfactants can increase the solvent capacity for the drug. It can also provide flexibility to interfacial films by reducing the bending stress at interface and evolve different curvatures necessary for formation of nano-emulsion [25]. Our results represented clove oil/Tween 80/PG combination successful with less no of inversions and highest % transmittance of having clear appearance while ethyl oleate/Tween 80/PG 400 combinations showed the highest number of inversions and least % transmittance of 31.24 ± 0.58% with turbid appearance. So, we selected PG as the co-surfactant because of its highest emulsification potential for chosen oil and surfactant, its low HLB value and its previous use in much research.

### 2.3. Construction of Pseudo-Ternary Diagram

**Figure 2:**
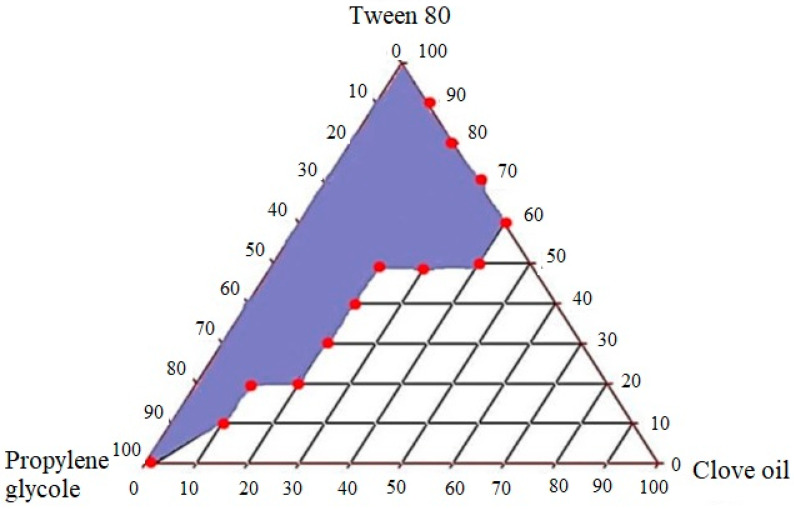
Construction of pseudo-ternary phase diagram with clove oil, Tween 80 and PG.

A ternary phase diagram was constructed after determining the solubility of the drug in different excipients. The objective of this step was to determine appropriate concentrations of oil, surfactant and co surfactants which can form clear nano-emulsions with self-emulsifying ability. Clove oil, tween 80 and PG were used for the construction of the ternary phase diagram (Figure 2). The dilution method was utilized for the preparation of samples and water was used as a constant factor and added up to 10 mL in each sample. According to the results, a wider nano-emulsion region was obtained, which is shown as shaded region in triangle. The oil percentage used was 10–90% for the preparation of different ternary mixtures. It was observed that clear systems were obtained with up to 40% of oil, while the use of more than 40% of oil produced turbid systems which can be due to increase in droplet size [26]. Increased oil concentration (˃40%) caused turbidity of emulsions, while the larger concentration of surfactant caused a decrease in turbidity. An increased amount of surfactant caused a more clear system because the surfactant provides stabilization by reducing the oil content at interface [27]. The use of less than 40% of surfactant caused instability in the system, resulting in phase change. The results depicted that wide nano-emulsion region was achieved by the use of essential oils, namely clove oil, Tween 80 and PG. Tween 80 has high HLB value 15 and formed stable nanoemulsifying formulations with clear appearance because high HLB value surfactants are required to form stable oil in water nano-emulsions [24]. The use of high HLB value surfactant Tween 80 with low HLB value co-surfactant PG produced greater nanoemulsifying region. These findings are in accordance with the study reported earlier by Elnaggar et al.

### 2.4. Preparation of CFX-Loaded SEDDS

Based on the pseudo phase diagram, four different amounts of ternary mixtures were chosen from the nano-emulsion region to prepare CFX-loaded SEDDS. As illustrated in Table 2, different concentrations of oils, surfactants and cosurfactants were utilized to prepare optimized SEDDS formulations. The process of preparing the SEDDS concentrate was by simple admixture of the ingredients and the solubility of the drug in the mixture was performed at room temperature. This ease of preparation supports the core theme of the project from industrial manufacturing and while scaling-up, same can be achieved by a planetary type mixer followed by homogenization.

### 2.5. Analysis of Drug Content

All formulations showed high drug entrapment of more than 90 % as shown in Table 3. The final amount (*w*/*v*) of CFX loaded into SEDDS was calculated to be 120 mg/mL, which can be due to the better mixability of drug with clove oil. It could serve as partial lipid phase, and similar results were obtained earlier [28] by using essential oils for SEDDS preparation.

### 2.6. Self-Emulsification Test

Emulsification rate is a key consideration for the characterization of effectiveness of self-emulsification [29]. We performed the self-emulsification to measure the emulsification time in which CFX-loaded SEDDS dispersed completely when undergoing dilution with mild agitation [30]. F2, F3 and F4 formulations dispersed quickly and completely in <1 min, as can be seen in Table 4. This can be described by the fact that the rise in concentration of surfactant can decrease interfacial tension, diffusion of water in oil phase by producing interfacial disruption and release of globules in aqueous medium that leads to quick dispersion of CFX-loaded SEDDS under gentle agitation. F1 formulation showed an emulsification time of >1 may be due to little amount of surfactant as compared to other formulations. However, the visual observations of formulations indicated no phase change. On the basis of these results, we can assume that, upon dispersion in GI fluids, these formulations will remain as SEDDS.

### 2.7. Cloud Point Measurement

Cloud point measurement is one of the key parameters in SEDDS formulations, especially those with non-ionic surfactants. Factors such as excipients amount, Smix ratios and drug hydrophobicity affect cloud points. Sudden cloudiness occurs when the cloud point is reached in a formulation. When cloud point is achieved, a further increase in temperature can cause phase separation in formulations containing nonionic surfactants which is due to the dehydration of poly ethylene oxide and can affect the drug absorption [31]. The cloud point of a formulation must be known in order to avoid the phase separation, and it should be greater than 37 °C. All formulations from F1 to F4 exhibited a cloud point greater than 37 °C, which means they will remain stable in vivo [30].

### 2.8. Robustness of SEDDS to Dilution 

The main objective of robustness to dilution studies of CFX-loaded SEDDS was to examine the uniformity of emulsions and any drug recrystallization which can occur during higher dilutions or upon storage for long time. The reason for using different media was to mimic different conditions of stomach and intestine in vivo. F2, F3 and F4 formulations remained stable in all media, which showed their robustness to dilution, and which suggested the drug will release uniformly in vivo [32]. Formulation F1 showed stability in distilled water, while it was unstable in other mediums, as shown in Table 5, which can be attributed to an increased amount of oil and lesser concentration of surfactant in comparison to other formulations [33].

### 2.9. Determination of Droplet Size, Zeta Potential and PDI

An important factor in the performance of self-emulsification is droplet size because it is directly related to drug absorption and helps in the measurement of the rate and extent of drug release. Emulsion droplets which are smaller in size cause rapid absorption and enhance the drug bioavailability [34]. Drug-loaded oily droplets with different concentrations of excipients displayed a difference in mean droplet size, as shown in Table 6. Formulations F2, F3 and F4 formulation exhibited a decrease in the droplet size compared to F1, which might be due to the lower concentration of oil and higher concentrations of the surfactants. The reason for low droplet size was the presence of the appropriate amount of surfactant, which causes adsorption. They create interfacial film by closely packed arrangement, which leads to a decrease in the size of droplets, while F1 showed greater droplet size than rest of the formulations due to greater concentration of oil and reduced surfactant concentration [35]. However, all CFX-loaded SEDDS formulation F1-F4 showed particles in nano emulsion range <100 nm. F2 showed the smallest droplet size and it was statistically significant, compared to the other formulations (*p* < 0.05). With regard to the zeta potential, however, formulation F1 showed a zeta potential of −1.96 ± 3.65, while F2 showed a zeta potential of −1.77 ± 5.09, and F3 and F4 showed a zeta potential of −2.47 ± 5.54 and −0.924 ± 6.98, respectively. Negative values of the zeta potential showed the presence of nonionic surfactants and, sometimes, the presence of free fatty acids. It was also reported that intestinal cells exhibited negative charge due to the presence of mucosal fluid, in order for positive droplets to provide better interactions with GI mucosa. Thus, at physiological pH, this formulation would reach a positive zeta potential [36]. Generally, it is considered that charged particles with high zeta potential values do not aggregate due to electrostatic repulsion, but this rule is not always strictly followed for systems with nonionic surfactants. 

Non-ionic surfactants are steric stabilizers which decrease zeta potential of formulations during their absorption due to a shift in the shear plan of particles. These steric interactions usually cause repulsion between particles which, thus, stabilize the system [37]. The final verdict about these values can only be made by performing long term stability studies. Higher value of PDI displays low uniformity of droplet size and vice versa. A PDI value of more than >0.7 shows broad distribution and non-uniformity of droplets [38]. In our study, all formulations exhibited PDI values < 0.4, which indicate that all SEDDS formulations (F1–F4) have narrow size distribution and uniformity in the size of droplets.

### 2.10. Stability Studies at Accelerated Conditions

Stability of nano emulsion is a key parameter that ensures that all SEDDS formulations will be stable in all conditions. CFX-loaded SEDDS formulations F2, F3 and F4 showed no phase change, phase separation or any other instability issue, while F1 showed minor phase change. This can be due to an appropriate amount of oil and Smix which helps in preventing thermodynamic instability by inhibiting the agglomeration of droplets through forming strong barriers and reducing free energy. A surge in the concentration of oil which was 30% and decrease in Smix ratio cause instability in F1 formulation [33]. Owing to the above findings that F1 is not stable under the tested circumstances, it was discontinued for further testing. Accelerated chemical stability studies conducted for the period of 12 weeks demonstrated the slight decrease in active contents of CFX and clove oil, observed when stored at higher temperature 45 °C. Due to the oxidation process during storage at higher temperature 45 °C, clove oil may result in degradation. On the other hand, all formulations showed no sign of degradation and remain stable when stored at 4 °C [2].

### 2.11. In Vitro Dissolution Studies

**Figure 3:**
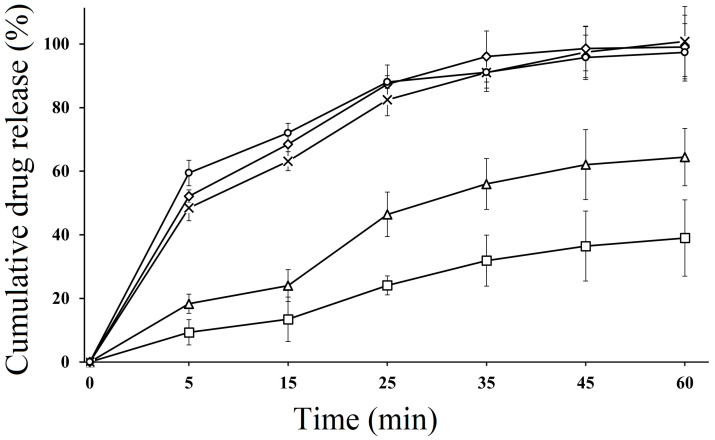
In vitro dissolution study of pure CFX P1 (□), marketed CFX suspension F5 (∆) CFX from oily droplets F2 (○), F3 (×) and F4 (◊) in SGF (0.1 N HCL, pH 1.2) at 37 °C for 60 min. Data are presented as means (*n* = 3) ± SD.

**Figure 4:**
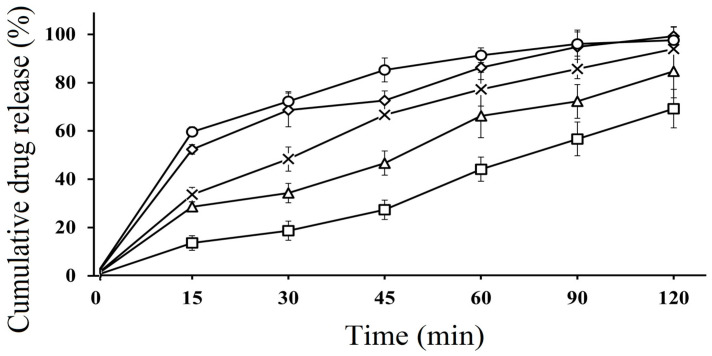
In vitro dissolution study of pure CFX P1 (□), marketed CFX suspension F5 (∆) CFX from oily droplets F2 (○), F3 (×) and F4 (◊) in SIF (PBS, pH 6.8) at 37 °C for 120 min. Data are presented as means (*n* = 3) ± SD.

In vitro dissolution studies of SEDDS formulations were performed in both acidic and basic media. The dissolution behavior of CFX-loaded SEDDS formulations F2, F3 and F4 was compared with pure CFX (P1) and marketed CFX suspension (F5), which were used as control. According to the results, SEDDS formulations exhibited an improved drug release behavior compared to pure CFX as well as marketed CFX suspension in both acidic and basic media. In acidic media, CFX-loaded oily droplets (F2, F3 and F4) displayed a cumulative CFX release of 97.32 ± 4.82%, 95.67 ± 5.23%, 94.23 ± 3.91%, respectively, within 60 min, whereas pure CFX showed 37.89 ± 0.41% and marketed CFX suspension exhibited 65.98 ± 16% release, as illustrated in Figure 3. However, in basic pH, F2 showed 97.12 ± 5.02, F3 showed 94.35 ± 4.75% and F4 showed 96.01 ± 5.42% of cumulative drug release, while pure drug P1 and marketed drug F5 showed a release rate of 38.43% ± 2.45% and 85.13% ± 3.23%, respectively, within 120 min, as depicted in Figure 4. The CFX release from all SEDDS formulations was significantly higher in both types of media as compared to P1 and F5. A spontaneous nano emulsion formation takes place due to the smaller droplet size resulting in an increase in rate and extent of dissolution [39]. Furthermore, the dramatic increase in the release rate of CFX from SEDDS formulations explain the rapid disperse ability of the drug and its ability to retain the drug in solubilized form, thus providing a higher dissolution rate [40]. These results also supported the hypothesis that the release of poorly water soluble drugs can be improved by use of nanosized droplets, while the slow release rate of plain drug and marketed drug can be attributed to poor water solubility [41]. Although there was a minimal difference between release rates of all SEDDS formulations in both media, the F2 formulation showed the highest release rate in both media. This behavior could be due to the presence of appropriate proportions of oil and surfactant in the system and because of the lowest droplet size. The good release profile of SEDDS formulations in acidic and basic conditions indicate that drug will release efficiently in the stomach and intestine, resulting in an improvement in absorption across the intestinal membrane followed by enhanced bioavailability.

### 2.12. Ex Vivo Permeation Studies

**Figure 5:**
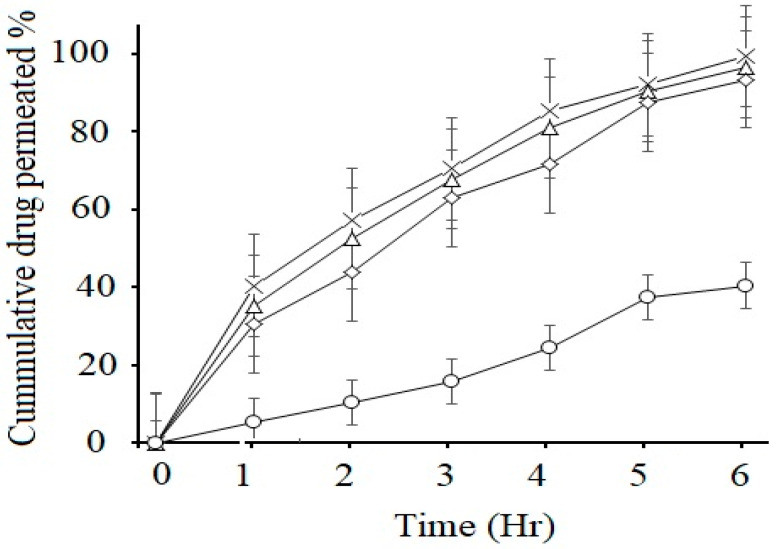
Ex vivo permeation studies of marketed CFX suspension F5 (○) and CFX from oily droplets F2 (×), F3 (∆) and F4 (◊) in PBS pH 6.8 at 37 °C. Data are presented as means (*n* = 3) ± SD.

Permeability of drug in GI tract is an important factor for the different dosage forms which will predict the absorption and bioavailability of the drug. The non-inverted rat intestinal sac method was used for the prediction of in vivo absorption of CFX. This method has several advantages over in vivo animal studies, such as less sampling is required and its experimental cost is also less. The results showed that there was a significant increase (*p* < 0.05) in intestinal permeability of CFX-loaded SEDDS formulations F2, F3 and F4 as compared to marketed suspension F5 (control), as illustrated in Figure 5. All lipid-based formulations exhibited higher permeation of the loaded drug compared to control. According to the results, within 6 h, F2 showed maximum drug permeation rate of 99.43 ± 7.05% and F3 showed 96.54% ± 5.43 and F4 showed 93.45 ± 4.32%, respectively, while marketed suspension showed only 37.43 ± 4.23%. The improved permeation of the drug from oily droplets was due to the usage of suitable concentrations of surfactants, which resulted in higher permeation across the membrane [42]. Another important factor for enhancement of permeation rate is incorporating CFX into SEDDS, which increases its absorption by retaining the drug in solubilized form at absorption sites. Enhancement in the permeation rate of CFX from SEDDS can be due to passive diffusion, pinocytosis or endocytosis, which is due to the absorption mechanism of the oil droplet interface [43]. In addition, nanosized droplets in SEDDS provide greater interfacial surface area for release of drug and increase in permeation process [44]. Ex vivo permeation from control marketed brand was low due to poor dissolution and less solubility in intestinal media, which hindered the passage of drug molecules from intestinal epithelial cells. Increased permeability through intestinal membrane would lead to increased concentration of the active drug into systemic circulation, which ultimately leads to improved bioavailability [3].

Apparent permeability coefficient P*_app_* was calculated by the given formula and permeability enhancement ratios obtained are shown below in Table 7. 

Overall, the biopharmaceutical parameters that are indicative of a higher bioavailability showed that loading CFX into the SEDDS is a promising strategy. On one hand, because of the oily nature and very small droplet size (5–10 folds smaller than the pores of mucus lining), SEDDS were able to permeate deeper into the mucosal lining and keep the drug in dissolved form and, on the other hand, after reaching the epithelial lining, they will be able to release almost all of the drug contents. Although the proof of the concept needs an in vivo investigation in future, it can be safely estimated that the strategy will provide a higher exposure of drug to microorganisms after improved absorption. With the same dose of CFX loaded into SEDDS, a higher Cmax and longer duration of action are expected. The improvement in these two parameters will tremendously enhance the therapeutic efficiency of CFX against the *Staphylococcus aureus* where a higher MIC is involved and, therefore, the chances of microbial resistance will be decreased. 

## 3. Material and Methods

### 3.1. Material

Cefixime (CFX) was donated from Global Pharmaceutical Industry, Ltd. (Islamabad, Pakistan). Tween 80 (polyoxy ethylene sorbitan monooleate) and Span 80 (sorbitan monooleate) were obtained from Sigma Aldrich, Germany. Caproyl 90 (propylene glycol monocaprylate) was purchased from Gattefosse, Lyon, France. Kolliphor EL (polyoxyl 35 hydrogenated castor oil), Propylene glycol (1,2-propanediol) and PEG 400 (polyethylene glycol) from VMR Chemicals, (UK). Oleic acid (cis-9-octadecenoic acid), Miglyol 812 (caprylic/capric triglyceride), clove oil and ethyl oleate were purchased from Aladdin Industrial Corporation, Shanghai, China. All chemicals were of analytical grade and so, were used without additional purification.

### 3.2. Methods

#### 3.2.1. Screening of Various Excipients Based on Solubility

Solubility studies of CFX were carried out in different oils, including oleic acid, clove oil, ethyl oleate and miglyol 812, by adding the increasing amount of drug in glass vials with 5 mL of each vehicle. The mixture was blended at 37 ± 0.5 °C for 72 h and subjected to ultra-sonication for 2–3 min. Afterward, the mixtures were centrifuged at 14,000 rpm for 5 min. Filtration of the supernatant was carried out through PTFE membrane filter containing the pore size of 0.45 μm. The amount of the loaded drug was measured by diluting the supernatant with methanol. The concentration of drug loaded was measured with the help of UV spectrophotometer at 288 nm (V-530; JASCO Corporation, Tokyo, Japan). Each experiment was performed in triplicate [29].

#### 3.2.2. Assortment of Surfactants and Co-Surfactants Based on Emulsifying Ability

To arrange various surfactants and co-surfactants based on emulsification capability, 300 mg of each surfactant (Tween 80, Span 80 and kolliphor EL) was mixed with 300 mg of oil phase (clove oil, oleic acid, ethyl oleate and miglyol 812). For homogenization and through mixing, the mixtures were warmed at 60 °C and centrifuged. After the homogenization of mixtures, 50 mg of each mixture was diluted up to 10 mL with de-ionized water to form nano-emulsion by flask inversion method. Nano-emulsions were evaluated visually for any kind of turbidity. Subsequently, the transmittance of nano-emulsions was determined after 2 h at 638.2 nm with the help of UV spectrophotometer (V-530; JASCO Corporation, Tokyo, Japan).

Co-surfactants were also screened for their capability to promote the emulsifying potential of surfactants in the same manner as mentioned above, except for the variation in the quantities. To evaluate co-surfactant, 300 mg of oil, 200 mg of surfactant and 100 mg of co-surfactant were used [25].

#### 3.2.3. Preparation of Pseudo Ternary Phase Diagram

Ternary phase diagram was prepared to categorize the self-emulsifying regions. For this purpose, large numbers of ternary mixtures were prepared with different compositions of selected oils, surfactants and co-surfactants. These excipients were combined to make a total of 100% of the final formulation. Each formulation, with varying concentrations of components, was prepared and then, homogenization was carried out with stirring at 300 rpm at 40 °C for 5 min. The resulting formulation was then diluted with 10 mL distilled water and observed visually. Pseudo ternary phase diagram was constructed usingSigmaPlot^®^ software (version 13) [45].

#### 3.2.4. Development of CFX-Loaded SEDDS

The pre-evaluated combination mixture of surfactant oil and co-surfactant was homogenized by continuous stirring at 150 rpm at 25 °C. A total of 100 mg of the drug was incorporated into the SEDDS under continuous stirring until the development of a transparent emulsion mixture. The resulting drug-loaded SEDDS formulations were stored at 25 °C for further use [46].

#### 3.2.5. Physicochemical Characterization of SEDDS

##### Drug Content Analysis

In order to determine drug content, CFX was extracted from SEDDS by diluting one part of CFX-loaded SEDDS pre-concentrate with nine parts of methanol. This mixture was centrifuged for 10 min at 15,000 rpm. Supernatants were then filtered through PTFE membrane filter of 0.45 µm pore size. Drug content was estimated at 288 nm with the help of UV spectrophotometer by using methanol as blank [47].

##### Determination of Self-Emulsification Time 

Self-emulsification time on dilution with aqueous phase was calculated by adding 0.02 g of CFX-loaded SEDDS to 200 mL of 0.1 N HCL (pH 1.2) under continuous stirring at 50 rpm at 37 °C. Visual assessments of the formulations were carried out in order to evaluate the time required to form a homogenous mixture or complete disappearance of pre-concentrates [48].

#### 3.2.6. Robustness of SEDDS to Dilution 

The robustness of CFX-loaded SEDDS formulations was studied by diluting 50, 100 and 1000 times in different media and stored for 24 h. Thereafter, the samples were observed for phase change. Dissolution media used were de-ionized water, 0.1 N HCL and Phosphate buffer with a pH of 6.8 [49].

#### 3.2.7. Evaluation of Droplet Size, Zeta-Potential and Polydispersity Index 

The size, zeta potential and PDI of drug-loaded SEDDS were evaluated by Zetasizer ZS 90 (Malvern Instruments, Malvern, UK). Nanoemulsions were prepared by diluting CFX-loaded SEDDS pre-concentrate at a ratio of 1:100 with d-ionized water. The analysis was carried out at 25 °C [50].

#### 3.2.8. In Vitro Drug Release Studies

SEDDS formulations were evaluated for drug release by using dialysis membrane tubes with the molecular weight cut off 14 kDa, (Sigma Aldrich, St. Louis, MO, USA) in USP type II dissolution apparatus (Galvano Scientific, Lahore, Pakistan). Prior to the experiment, the dialysis membrane was immersed in mixture of tween 20 and de-ionized water for 24 h. At the time of the experiment, CFX-loaded SEDDS formulations (2 mL) were sealed in dialysis tubes. These dialysis tubes were then placed in beakers containing 300 mL of release buffer medium (simulated gastric fluid pH 1.2 and simulated intestinal fluid pH 6.8) and the whole system was stirred at 100 rpm at physiological conditions. An aliquot of 2 mL was removed at fixed time points (0, 15, 30, 60 min), while replacing it with the equal amount buffer medium. Afterward, aliquot parts were passed through membrane filters with a pore size of 0.45 um and the amount of the released drug content was evaluated at a wave length of 288 nm using Shimadzu 1280, UV-spectrophotometer NY, USA [51]. Sensitivity and accuracy of UV-spectrophotometer was verified and confirmed by taking the absorption of different known concentration of standard solutions of CFX at wavelength of 288 nm. Initially, calibration curve was plotted against gradual increasing concentration of standard CFX solutions and then, absorption of known solutions was evaluated [49].

#### 3.2.9. Storage Stability Studies

##### Thermodynamic Stability Studies 

Thermodynamic stability studies of optimized formulations were carried out to evaluate their stability by following three methods.

##### Heating and Cooling Cycle

Heating and cooling cycle test was performed for optimized formulations for 48 h, with six cycles at 40 °C and 4 °C, respectively. Afterward, drug precipitation and phase separation in formulations were observed. The SEDDS preparations which passed the test were taken for centrifugation test.

##### Centrifugation Study 

The formulations which remained stable during heating and cooling cycle test were exposed to centrifugation test at 14,000 rpm for 30 min. Preparations were then examined for instability issues such as creaming and phase separation. Stable formulations were used further for application of freeze thaw cycle.

##### Freeze Thaw Cycle 

Three freeze thaw cycles of the drug-loaded SEDDS formulations were carried out by freezing at −21 °C for 24 h and then, thawing at 25 °C for 24 h. Oily droplets were then examined for any instability issue. Only stable formulations were selected for further investigations [52].

##### Chemical Stability Study

The chemical stability of the selected formulations was evaluated by storing the formulations at three different temperatures; 4 °C, 30 °C and 45 °C, for a duration of 12 weeks. The chemical stability of CFX and other excipients was evaluated at the end of the storage time. Clove oil was especially analyzed, as it was reported to have undergone oxidative stress during storage conditions. The analysis of CFX and excipients was performed by UV spectrophotometer, by using a method previously described with slight modifications [1].

#### 3.2.10. Measurement of Cloud Point of SEDDS

To determine the cloud point of CFX-loaded SEDDS, formulations were diluted (1:100) with de-ionized water. Thereafter, the formulations were placed in a water bath at 37 °C and temperature was augmented slowly until the appearance of cloudiness. The temperature at which sudden cloudiness appeared was defined as cloud point [53].

#### 3.2.11. Ex Vivo Permeation Study

For the assessment of enhanced drug permeability of SEDDS formulations, ex vivo permeation studies were performed by using freshly excised rat intestinal membrane via non-everted intestinal sac method. 

For this purpose, CFX-loaded SEDDS formulations and marketed suspension of CFX were compared by non-everted intestinal sac technique. Institutional ethical approval was obtained from Research and Ethics Committee of Riphah Institute of Pharmaceutical Sciences, Islamabad, Pakistan. Ex vivo permeation studies were carried out according to the guidelines provided by National Institute of Health (NIH), Islamabad, Pakistan. Briefly, fasting Sprague Dawley male rats weighing between 250 and 300 g were sacrificed. The duodenal part of the small intestine was removed, cut into pieces of 6 cm length and placed into the normal saline. The debris, mucus and luminal contents of the pieces were washed with saline solution. Control was prepared by diluting 50 mg of reconstituted CFX suspension with 1 mL of phosphate-buffered saline (PBS) pH 6.8. Sample solution was prepared by diluting CFX-loaded SEDDS formulations with 1 mL of PBS using equivalent amount of the CFX. The control formulations and the samples were injected into the lumen of duodenum while the drug leakage from the other side of the lumen was avoided by tightly closing it with a thread. Thereafter, each lumen of the duodenum was placed in different chambers of organ bath comprising 10 mL of PBS at 37 ± 0.5 °C with continuous aeration. At predetermined time intervals, aliquots of 3 mL from each sac were removed from the outer side and replaced with equal amount of pre-heated fresh medium at 37 ± 0.5 °C. The amount of drug permeation was estimated by UV-visible spectrophotometer as described above. The permeability of CFX was calculated by utilizing a plot of cumulative CFX permeation across the duodenum verses time [54]. Furthermore, apparent permeability was calculated by using following formula,

P*_app_* = Q/Act (1)

where P*_app_* is permeability coefficient, Q (mg)= total amount permeated through intestine, A (cm^2^) = surface area of permeation surface, C (mg/cm^3^) = initial concentration of drug, t = total time of permeation study.

#### 3.2.12. Statistical Interpretations

Statistical data analysis was carried out using DD-Solver (1.0) software. Student’s *t*-test and one way analysis of variance (ANOVA) was used for the evaluation of difference between data. *p* value < 0.05 used as a minimum level of significance. All the experiments were carried out in triplicate and results were described in mean ± standard deviation (SD).

## 4. Conclusions

A simple and easy-to-scale-up for industrial manufacturing approach of preparing CFX-loaded SEDDS was used in this study by using essential oil, Tween 80 and PG. CFX-loaded oily droplets displayed a mean droplet size < 100 nm and improved drug dissolution and permeation compared to pure CFX and marketed CFX suspension. Among all tested formulations, the optimized formulation F2 exhibited significant improvement in dissolution and intestinal permeation, approximately 2.71-fold higher apparent permeability coefficient as compared to the marketed drug. The findings of in vitro studies suggested that SEDDS are an effective carrier system for enhancing the oral bioavailability of CFX that, in turn, will provide better therapeutic efficacy and lower the chances of resistance.

## Figures and Tables

**Table 1:** Screening of Surfactants via Emulsification Test and % Transmittance.

Oil/Surfactant	UV Transmittance (%)	No of Inversions	Appearance
Ethyl oleate/Tween 80	98.33 ± 0.27	2	Clear
Clove oil/Tween 80	96.29 ± 0.16	4	Clear
Ethyl oleate/Span 80	62.15 ± 2.32	>20	Turbid
Clove oil/Span 80	69.32 ± 0.11	>20	Turbid
Ethyloleate/KolliphorEL	93.57 ± 0.39	3	Clear
Clove oil/KolliphorEL	91.51 ± 0.13	4	Turbid

**Table 2:** Composition of optimized SEDDS formulations.

Name	CFX (mg)	Clove Oil (%)	Tween 80 (%)	PG (%)
F-1	100	30	50	20
F-2	100	10	70	20
F-3	100	20	60	20
F-4	100	20	50	30

**Table 3:** Drug content of optimized SEDDS formulations (mean ± SD, *n* = 3).

Formulations	Drug Content (%)
F-1	91.21 ± 4.65
F-2	96.32 ± 5.34
F-3	95.21 ± 2.56
F-4	92.54 ± 7.25

**Table 4:** Emulsification and cloud point measurements of Cefixime-loaded SEDDS formulations.

Formulation Code	Self-Emulsification Test (Sec)	Visual Observation	Cloud Point Measurement (°C)
F-1	>1	No phase change, Clear	77–79
F-2	40.37 ± 3.0	No phase change, Clear	82–84
F-3	43.95 ± 2.0	No phase change, Clear	79–81
F-4	47.66 ± 2.0	No phase change, Clear	76–78

**Table 5:** Robustness to dilution studies of cefixime-loaded SEDDS formulations in different media.

Formulation’s Code	De-Ionized Water	0.1 N HCL pH (1.2)	PBS pH (6.8)
F-1	stable	unstable	unstable
F-2	stable	stable	Stable
F-3	stable	stable	Stable
F-4	stable	stable	Stable

**Table 6:** Estimation of droplet size, zeta potential and PDI of SEDDS formulations (mean ± SD, *n* = 3).

Formulation Code	Particle Size (nm)	Zeta Potential (mV)	Polydispersity Index (PDI)
F-1	87.33 ± 4.23	−1.96 ± 3.65	0.26
F-2	19.01 ± 1.12	−1.77 ± 5.09	0.20
F-3	20.16 ± 2.41	−2.42 ± 5.54	0.36
F-4	27.68 ± 2.41	−0.924 ± 6.98	0.24

**Table 7:** Apparent permeability of cefixime across the rat intestinal membrane and enhancement ratio of F-2, F-3, and F-4 self-nano emulsion formulations compared with F-5 marketed suspension. (Mean ± SD, *n* = 3).

Formulations/Control	Apparent Permeability Coefficient (P*_app_* × 10^−5^ cm/s)	Ratios of Apparent Permeability Enhancement
F-2	9.02 ± 1.23	2.71
F-3	8.75 ± 1.56	2.63
F-4	8.48 ± 1.05	2.55
F-5 marketed suspension (control)	3.32 ± 0.74	-

## Data Availability

Not applicable.
